# Gender differences in workplace violence against physicians of obstetrics and gynecology in China: A questionnaire in the national congress

**DOI:** 10.1371/journal.pone.0208693

**Published:** 2018-12-10

**Authors:** Lan Zhu, Lei Li, Jinghe Lang

**Affiliations:** Department of Obstetrics and Gynecology, Peking Union Medical College Hospital, Peking Union Medical College, Beijing, China; Università Cattolica del Sacro Cuore, ITALY

## Abstract

**Background:**

China has witnessed a surge in violence against medical personnel, including widely reported incidents of violent abuse, riots, attacks, and protests in hospitals, but little is known about the impact of gender differences on the workplace violence against physicians of obstetrics and gynecology. The aim of this study was to analyse gender differences in workplace violence against physicians of obstetrics and gynecology in China.

**Methods:**

Printed questionnaires were sent to participants of a national congress of obstetricians and gynecologists. The questionnaire consisted of items relevant to epidemiologic characteristics, workplace violence experienced in the past 12 months, participants’ attitudes toward violence and physician–patient relationship. Data from female and male physicians were compared in univariate and multivariate analyses.

**Results:**

We sent out 1,425 questionnaires, and 1,300 (91.2%) physicians responded. Among 1,247 participants with specified gender, female and male physicians consisted of 162 (13.0%) and 1,085 (87.0%), respectively. Over the past 12 months, about two-thirds of these physicians suffered verbal abuse in the workplace, gender difference aside. After adjustment for education status, working hospital and subspecialty, male physicians had suffered more physical assaults than female colleagues (18.8% vs. 10.5%, adjusted odds ratio [OR] 2.3, 95% confidence interval [CI], 1.4–3.7), most attacks without apparent physical injuries (adjusted OR 2.3, 95% CI, 1.4–3.7). Male physicians also suffered more sexual assaults than female colleagues (5.0% vs. 1.3%, adjusted OR 4.8, 95% CI, 1.8–13.3), nearly all of verbal harassment. There were only two sexual attacks on female physicians, and no rapes occurred. Although almost all physicians regarded the current circumstance as “unhealthy and stressful”, more than half of them would take various active initiatives to create and maintain healthy and friendly physician–patient relationships.

**Conclusion:**

Male physicians of obstetrics and gynecology in China suffered the same number of verbal abuse incidents but more physical and sexual assaults than their female colleagues. Both genders had similar opinions about causes, consequences and management about work violence against physicians, and had the same pessimistic perspectives but innovative wishes for the physician–patient relationship.

## Introduction

Over the past few years, China has witnessed a surge in violence against medical personnel, including widely reported incidents of violent abuse, riots, attacks, and protests in hospitals. However, incidents of violence against medical personnel have increased in intensity. [1,2,3] According to a survey by the Chinese Hospital Association, incidence of verbally abusive language and threats to medical workers increased from 90% in 2008 to 96% in 2012, whereas the incidence of physical injuries of medical workers escalated from 47.7% in 2008 to 63.7% in 2012. The average annual number of assaults on medical staff per hospital increased from 20.6 assaults in 2008 to 27.3 assaults in 2012. [4] Stressful physician–patient relationships even contributed a novel word, “Yi Nao”, meaning medical or hospital disturbance. [5] Most events of Yi Nao consisted of severe violence against medical staff, making the government worry about the situation and take measures. [5,6] Although many studies explored risk factors relevant to workplace violence, little is known about risk factors for physicians of obstetrics and gynecology, a specialty just dealing with female patients.

The national congress provided an opportunity to survey such issues with sufficient sample capacity, because most participants came across various regions of China. At the 11th National Academic Congress of Obstetrics and Gynecology (Congress), we initiated a questionnaire survey among registered physicians of obstetrics and gynecology to analyse patterns and risk factors of workplace violence against physicians.

## Materials and methods

### Overview

The Institutional Review Board of Peking Union Medical College Hospital (PUMCH) had approved this study. At the 11th Congress, convened by the Chinese Medical Association in Xiamen City, Fujian Province, China, from March 12 to 15, 2015, printed anonymous questionnaires were sent to and retrieved from all participating physicians registered as obstetricians and gynecologists. We collected and recorded completed data from returned questionnaires with enough information to use for the study. (At least two-thirds of the items were filled out.)

### Study population

Participants came across the country of China. They registered the Congress by means of on-line or post registration forms, and their certification as obstetricians and/or gynecologists were identified and confirmed by submitted materials to the Congress. At check-in reception of the Congress, we sent out questionnaires to every participant, and asked for on-site retrieve to ensure proper corresponding rate. We also collected questionnaire on the next day on the opening ceremony, or by mail of paid postage from participants who couldn’t fillout the form in time.

### Questionnaire design

The questionnaire was constructed by the staff of the Department of Obstetrics and Gynecology, PUMCH. We developed the questionnaire based partly on existing questionnaires [7,8,9,10,11,12,13,14,15,16,17,18] with some items added and modified to specifically reflect the Chinese setting. [19] The questionnaire was constructed by Lei Li and Lan Zhu. For validation, a preliminary study was conducted among 55 physicians of Department of Obstetrics and Gynecology in PUMCH. After discussion with the participants and researchers, the final version of the questionnaire was modified and approved with a total and separate Cronbach α>0.600 and Kaiser-Meyer-Olkin (KMO) measures>0.700. None of the 55 physicians validating the questionnaire participated in the study. The questionnaire consisted of 20 items: seven relevant to participants’ epidemiologic characters, four to workplace violence experienced in the past 12 months, nine to participants’ attitudes toward violence and the physician–patient relationship. In our study, workplace violence refers to any verbal abuse, physical assaults, or sexual assaults the physicians experienced over the past 12 months. Attitudes toward violence include causes, responsibilities, consequences and management of workplace violence against physicians, and perspectives and initiatives toward the physician–patient relationship. All questionnaires were check by Dr L Li and Dr L Zhu. Data were included only if at least two thirds items about participants attitudes toward workplace violence were specified.

### Statistical considerations

We collected data using Microsoft Excel tables. Statistical analyses were carried out using SPSS statistical software (version 19.0, SPSS Inc., Chicago, IL). Comparisons were made of categorical variables applied with a nonparametric κ^2^ test or Fisher’s exact test. Multiple-parameter analyses were performed using binary logistic analysis, calculating odds ratios (OR) and 95% confidence intervals (95% CI) to adjust for confounding factors.

## Results

### General characters of participants

We sent out 1,425 questionnaires to all the attendee, and 1,300 physicians (91.2%) responded. All the responding participants were registered physicians of obstetrics and gynecology across China. Of 1,247 participants with specified gender, 162 (13.0%) were male physicians, and 1,085 (87.0%) were female physicians. Compared with male colleagues, fewer female physicians worked in tertiary hospitals or were engaged in the practice of gynecology rather than in obstetrics, whereas female physicians had longer working years (Table 1).

**Table 1:** Participants’ epidemiologic characteristics.

	Male physicians	Female physicians	P
**Mean age ± SD (years)**	**41.4±9.4 (153)**	**43.2±9.0 (1039)**	**0.074**
**Areas of workplace**			**0.064**
North and Northeast China	35.2% (50/142)	40.2% (410/1020)	
East and South China	43.0% (61/142)	42.2% (430/1020)	
Central China	15.5% (22/142)	15.2% (155/1020)	
Northwest and Southwest China	6.3% (9/142)	2.5% (25/1020)	
**Education status**			**<0.001**
Undergraduate	34.4% (55/160)	59.1% (628/1063)	
Graduate	65.6% (105/160)	40.9% (435/1063)	
**Working hospitals**			**0.001**
Tertiary hospital	87.6% (141/161)	75.5% (815/1079)	
Other medical services	12.4% (20/161)	24.5% (264/1079)	
**Working years**			**0.006**
<1 year	6.2% (10/162)	4.4% (47/1080)	
1–5 years	13.6% (22/162)	7.2% (78/1080)	
6–10 years	13.6% (22/162)	8.2% (89/1080)	
11–15 years	10.5% (17/162)	11.5% (124/1080)	
16–20 years	13.0% (21/162)	14.5% (157/1080)	
>20 years	43.2% (70/162)	54.2% (585/1080)	
**Subspecialty**			**<0.001**
Gynaecology	72.3% (115/159)	47.0% (501/1066)	
Obstetrics	8.2% (13/159)	21.0% (224/1066)	
Reproduction	1.3% (2/159)	4.0% (43/1066)	
General	18.2% (29/159)	28.0% (298/1066)	
**Professional title**			**0.272**
Chief physician	35.8% (58/162)	39.5% (427/1080)	
Associate chief physician	26.5% (43/162)	30.6% (330/1080)	
Attending physician	19.1% (31/162)	15.6% (168/1080)	
Resident physician	7.4% (12/162)	4.4% (48/1080)	
None or ambiguous profession title	11.1% (18/162)	9.9% (107/1080)	

### Prevalence of workplace violence

About four-fifths of physicians did not feel respect from their patients, and in this, there was no difference between female and male physicians (80.1% vs. 78.4%, P = 0.619, Table 2). About two-thirds of these physicians suffered verbal abuse in the workplace without gender difference (female vs. male, 62.2% vs. 66.7%, P = 0.277). For both genders, blame was the most common verbal abuse (female vs. male, 60.1% vs. 64.5%, P = 0.289), followed by verbal threats to security and property (30.4% vs. 38.0%, P = 0.073). Frequency of verbal abuse was distributed equally to both genders (Table 1).

**Table 2:** Respect and violence against participants in workplace.

	Male physicians	Female physicians	P
**Feeling respect from patients**	**21.6% (35/162)**	**19.9% (214/1074)**	**0.619**
**Verbal abuse**	**66.7% (104/156)**	**62.2% (657/1057)**	**0.277**
Blame	64.5% (100/155)	60.1% (630/1049)	0.289
Blame ≥4 times/12 months	19.0% (19/100)	20.3% (128/630)	0.760
Verbal threats	38.0% (52/137)	30.4% (282/929)	0.073
Threats ≥4 times/12 months	15.4% (8/52)	16.7% (47/282)	0.819
**Physical assault**	**18.8% (27/144)**	**10.5% (99/947)**	**0.004**
Without apparent physical injuries	16.2% (23/142)	9.5% (89/941)	0.014
With mild physical injuries	7.2%% (10/139)	4.4% (41/925)	0.155
With moderate physical injuries	2.2% (3/137)	2.3% (21/915)	1.000
With severe physical injuries	2.2% (3/136)	2.0% (18/905)	0.748
Assaults ≥4 times/12 months	0.7% (1/144)	1.6 (15/947)	0.710
**Sexual assaults**	**5.0% (7/140)**	**1.3% (12/915)**	**0.008**
Sexual harassment	5.0% (7/140)	1.1% (10/914)	0.004
Sexual attacks	0	0.2 (2/911)	1.000
Rapes	0	0	-
Assaults ≥4 times/12 months	1.4% (2/140)	0.3 (3/915)	0.133

Male physicians suffered more physical assaults than female physicians did (18.8% vs. 10.5%, OR 2.0, 95% CI, 1.2–3.1, P = 0.004). After adjustment for education status, working hospital and subspecialty, gender remained an independent risk factor for physical assault (adjusted OR 2.3, 95% CI, 1.4–3.7, P = 0.001).The most common physical assaults were those without apparent physical injuries (adjusted OR 2.0, 95% CI, 1.2–3.4, P = 0.001). Frequency of physical assault was distributed equally between both genders.

Male physicians suffered more sexual assaults than female physicians did (5.0% vs. 1.3%, OR 4.0, 95% CI, 1.5–10.2, P = 0.008). After adjustment for education status, working hospital and subspecialty, gender remained an independent risk factor for sexual assault (adjusted OR 4.8, 95% CI, 1.8–13.3, P = 0.002). Most assaults were verbal harassment (adjusted OR 6.3, 95% CI, 2.2–18.2, P = 0.001). Only two female physicians reported definite physical attacks and no rapes occurred.

### Participants’ attitudes toward workplace violence

With regard to participants’ attitudes toward workplace violence and physician–patient relationship, some differences of gender in univariate analysis (Table 3) had no significance after adjustment for education status, working hospital and subspecialty of physicians. In general, more than half of the participants considered “patients’ expectations difficult to meet” (85.2%) and “adverse media reports” (87.1%) as external causes of workplace violence and stressful physician–patient relationships, but “insufficient communication of skills” (74.5%) and “undesirable service awareness” (62.4%) as physicians’ responsibilities. Psychological stress (90.3%) and destruction of medical practice (79.5%) were the most common negative impacts of workplace violence. Most physicians (75.3%) expected to resolve violent events through the legal process, but actually most (78.4%) sought help from hospital administration. More than half of the physicians (53.5%) were pessimistic, and less than a tenth (6.5%) were optimistic about future physician–patient relationships. Almost all physicians (99.0%) regarded current physician–patient relationships as “unhealthy and stressful”, even though more than half of the physicians would take various active initiatives in creating and maintaining healthy and friendly physician–patient relationships (Table 3, “Agreeing with following ideas and practice related to physician–patient relationship”).

**Table 3:** Participants’ attitudes toward workplace violence and physician–patient relationship.

	Male physicians	Female physicians	P
**External causes**			
Patients’ expectations difficult to meet	77.2% (125/162)	86.9% (939/1080)	0.001
Adverse media reports	79.6% (129/162)	88.0% (950/1080)	0.003
Difficulty and too expensive to see a doctor	29.0% (47/162)	35.8% (387/1080)	0.090
Outdated health service	17.9% (29/162)	25.3% (272/1076)	0.041
**Physicians’ responsibilities**			
Insufficient communication of skills	71.0% (115/162)	75.1% (811/1080)	0.263
Brokerage, bribe and other financial temptation	16.0% (26/162)	22.8% (246/1080)	0.053
Undesirable service awareness	62.3% (101/162)	62.4% (671/1076)	0.997
Lack or insufficient skill training	37.7% (61/162)	35.9% (386/1076)	0.660
Undesirable responsibility or morality	32.1% (52/162)	34.9% (376/1076)	0.478
**Impact on physicians**			
Economic loss	49.4% (80/162)	35.0% (378/1080)	<0.001
Psychological stress	87.0% (141/162)	90.8% (981/1080)	0.127
Destruction of technology practice	73.5% (119/162)	80.0% (864/1080)	0.056
**Expected management of workplace violence**			
Negotiation with perpetrators	26.5% (43/162)	29.0% (313/1079)	0.518
Hospital administrations	30.2% (49/162)	45.1% (487/1079)	<0.001
Media exposure	9.9% (16/162)	4.4% (48/1079)	0.004
Lawsuit	78.4% (127/162)	75.1% (810/1079)	0.359
**Practical management of workplace violence experienced by self or colleagues**			
Submission to humiliation	11.1% (18/162)	13.8% (149/1078)	0.346
Negotiation with perpetrators	14.8% (24/162)	15.6% (168/1078)	0.801
Striking back on the scene	28.4% (46/162)	19.3% (208/1078)	0.007
Hospital administrations	69.8% (113/162)	79.5% (857/1078)	0.005
Media exposure	3.7% (6/162)	2.4% (26/1078)	0.295
Lawsuit	34.0% (55/162)	39.0% (420/1078)	0.221
**Features of current physician**–**patient relationship in China**			
Unhealthy and stressful	98.8% (160/162)	99.1% (1070/1080)	0.663
Healthy and friendly	1.2% (2/162)	0.9% (10/1080)	
**Expectation for future physician**–**patient relationship in China**			**0.088**
Optimistic	10.5% (17/162)	5.9% (63/1065)	
Neutral	44.4% (72/162)	46.2% (492/1065)	
Pessimistic	45.1% (73/162)	47.9% (583/1065)	
**Attitudes about children choosing medical career**			**<0.001**
Approval/neutral	41.0% (66/161)	26.1% (288/1066)	
Reluctant	59.0% (95/161)	73.9% (788/1066)	
**Agreeing with following ideas and practice related to physician**–**patient relationship**			
Asking for family members being on the scene for emergencies	67.9% (106/156)	70.4% (736/1045)	0.528
Refusing brokerage and bribe	93.0% (146/157)	95.7% (1014/1060)	0.140
Explaining medical expenses in detail	54.8% (86/157)	63.3% (671/1060)	0.040
Responding to patients’ requirements whenever necessary	56.3% (89/158)	62.3% (658/1057)	0.154
Avoiding unnecessary intimacy with patients beyond common practice	92.4% (146/158)	90.9% (963/1059)	0.544
Considering everything from patients’ standpoint	84.2% (133/158)	90.4% (956/1058)	0.018
Giving palliative treatment to all patients	62.3% (96/154)	64.8% (666/1027)	0.544
Maintaining harmonious relationship with patients without improper remarks	91.7% (143/156)	94.1% (998/1061)	0.248
Familiar with every patient’s therapeutic process in detail	79.5% (124/156)	84.8% (894/1054)	0.089

## Discussion

In recent years, violence against physicians has occurred frequently in China due to various reasons, which have been widely reported by Chinese and international media. The *Lancet* published a couple of articles, one entitled “*Ending violence against doctors in China*” [2] and the other entitled “*Appeal from Chinese doctors to end violence*”. [3] In early 2014, the BBC produced two reports about violence against physicians in China, and the *Times Weekly* raised the question of “*Why China’s Doctors Are Getting Beat Up*”, illustrating that engaging in frontline medical care in China is becoming a nightmare and disclosing the increasingly harsh medical environment for Chinese medical staff. The intense physician–patient relationship and the frequent violence against doctors seriously damage the prospects for Chinese doctors. [10] Workplace violence against physicians is categorised as a type II assault; most common to the health care setting is a situation in which the perpetrator has a legitimate relationship with the business and becomes violent while being served by the business. [20,21] Episodes of workplace violence of all categories are grossly underreported, [7,12,22,23,24] which is due in part to specific health care culture. [25]

Reported workplace violence against physicians of obstetrics and gynecology is similar to a high prevalence in other studies in China. [4,8,26] Both doctors and nurses have been the targets of violent and distressing events, resulting in emotional pain, physical injury, and even death. [27] In studies from China, exposure to violence significantly affected emotional exhaustion, job satisfaction, and intention to leave, and the intensity of the harm from high-frequency exposure was several times stronger than that of low-frequency exposure. [18] After an episode of workplace violence, there are increased rates of damage to the physical and mental health, and economics among staff members. [28] Injuries associated with workplace violence result in longer work absences than other injuries. [25] Anxiety and depressive symptoms are common among physicians in China, and the issue of physician–patient relationship is particularly stressful. [17] These negative impacts on physicians probably distort whole situations of employment and quality of health care in China. Because doctors have low job satisfaction overall, recruitment and retention of doctors have become major challenges for the Chinese health care system. [9] Although all these findings are discouraging and upsetting, our study discovered that half of the participants would take various active initiatives in creating and maintaining healthy and friendly physician–patient relationships, such as “refusing brokerage and bribe”, “responding to patients’ requirements whenever necessary”, “maintaining harmonious relationship with patients without improper remarks” and so on (Table 3).Furthermore, many physicians reflected deeply about their responsibilities in workplace violence, and most regarded “insufficient communication of skills” and “undesirable service awareness” as important obstacles in healthy physician–patient relationships. These discussions and genuine deliberateness could help reveal and eventually resolve workplace violence.

Whether violence in gynecologists is different from violence in other categories of doctors remains imperative before proposing violence prevention measures. One-year prevalences of 6.2% and 13.9% were reported for physical and verbal aggressions toward healthcare workers from an infectious disease hospital. [29] Some 6.8% of radiologists in public hospitals experienced physical abuse in the previous 12 months. [30] One out of ten workers reported physical assault, and one out of three exposure to non-physical violence in the workplace in the previous year among health care workers in a Public Health Care Facility in Italy. [31] However, our study reported significantly higher prevalence of assaults than these reports, illustrating the severity of physician-patient relationship and necessity of comprehensive solution.

Few suggestions if any have supporting evidence to document their efficacy for reducing workplace violence. [28] Understanding physicians’ attitudes toward workplace violence and the physician–patient relationship may provide invaluable opportunities to repair stressful relationships and decrease violence against doctors, [1] especially in China. Problems arising from China should be eventually resolved when adapted to the Chinese context. Some authors promoted suggestions to address workplace violence in hospitals such as "improve physician–patient communication skills" and "preventive measures and plans" as major prevention methods, [32,33] because 93.0% of medical workplace violence was related to insufficient communication between hospital staff and patients. [14] However, patient dissatisfaction in China should be considered not as the cause of violence against doctors, but as a symptom of a flawed system that victimises both patients and doctors alike. As perceived by medical authority in China, fixing this damaged relationship is at the core of health-care reform. [34] When doctors are no longer made the scapegoats for the failures of health care reform, can we improve the physician–patient relationship and decrease the workplace violence in China, [35] which solution must come from steps to stimulate greater professionalism and rekindle trust between doctors and patients. [34]

Very few reports observed the pertinence of physician gender to workplace violence. Some found no differences in the probabilities of violence against female and male physicians. [11,19] However, both a Turkish national survey and a survey of resident doctors in New Zealand found verbal and sexual violence were seen more frequently among female physicians, whereas physical and economic violence were more frequent with male physicians. [13,16] The contrary conclusion came from a Japanese study, which found that verbal and sexual violence were seen more frequently among women, whereas physical and economic violence were more frequent with men, [15] but a Chinese survey of 2,464 medical professionals in 12 hospitals in two provinces discovered that male gender was one of the risk factors for workplace violence. [8] Causes of contradiction among these conclusions are unclear. Heterogeneity of physicians’ specialties may explain part of these paradoxical discoveries. In the field of obstetrics and gynecology, in which all patients are female, workplace violence against physicians may involve different factors than other situations. In our study, male physicians suffered more assaults than female colleagues, mainly from physical and sexual assault. It is not a surprising discovery. In traditional Chinese cultures, male physicians of obstetrics and gynecology have encountered much resistance and refusal from their patients, especially in underdeveloped cities. Female participants in our study, consisting of only13.0% of the population, may verify this cultural phenomenon indirectly. Cultural diversity probably cause behavioral and/or semantic confusion about the meaning of “violence” between female and male physicians. This aspect is of particular interest and significant because the difference in the rate of violence between the sex genders could be an indication of a gender gap. The greater prevalence of females than males in our sample is likely to reflect the gender gap described by Magnavita. [36] Our study provided primary data about gender discrepancies in this field. Furthermore, a traditional culture of repulsion to female doctors of obstetrics and gynecology should achieve transformation from positive media reports and delicate education of the public. It would not be easy but worthy of an active attempt. The differences of working years between female and male physicians would contributed the differences of violence distribution (Table 1), as previous studies indicate that age is important and that young doctors are more assaulted than older doctors. [30,37,38]

Selection bias was the most important drawbacks in our study due to the nature of the questionnaire survey and methods of sampling. According to data from the Chinese Obstetricians and Gynecologists Association, there were 190,062 registered physicians of obstetrics and gynecology as of December 31, 2007 (http://coga.org.cn/index). In this survey, 20906 (11.000%) physicians were male, approximating the ratio in our study (13.0%). Because of the growth in the physician cohort, participants in our survey represent less than 1% of the population. Although opinions and suggestions from participants are invaluable for direction of future clinical practice and health-care reform, caution is warranted when quoting and generalising the conclusions. Although the questionnaire provided free expressions for all items about attitudes toward violence and the physician–patient relationship, no physician responded with any supplement. A more delicate and detailed discussion about workplace violence probably needs more profound and time-consuming effort.

Recall bias was another drawback of our study. We had collected subjective statements without any possibility of verifying the true occurrence of violent incidents. The simplicity of the questionnaire used and the fact that the doctors came from various parts of China do not authorize the belief that all of them gave the term "violence" the same meaning, which is especially critical for the most delicate aspects, such as sexual violence. Even if the research is anonymous, the denunciation of having suffered sexual violence is never easy, which would influence the lower sexual violence reported by women compared to that of men.

In conclusion, male physicians in obstetrics and gynecology in China suffer identical verbal abuse but more physical and sexual assaults than their female colleagues. Both genders had similar opinions about causes, responsibilities, consequences and management of workplace violence against physicians and similar perspectives of the physician–patient relationship. Although nearly all physicians were pessimistic about current and future circumstances, most of them would take active initiatives to create and maintain healthy and friendly physician–patient relationships.

## Supporting information

S1 File Participant data. (XLSX)

S2 File STROBE checklist of this study. (DOCX)
